# Discrete-time survival analysis with survey weights: a case study of age at child death in Sierra Leone

**DOI:** 10.1186/s12889-023-16412-1

**Published:** 2023-08-10

**Authors:** Fundiswa Pearl Mdluli, Jesca Mercy Batidzirai

**Affiliations:** School of Mathematics, Statistics and Computer Science, University of KwaZulu-Natal, Pietermaritzburg, South Africa

**Keywords:** Discrete-time-to-event, Child mortality, OR-Odds Ratios

## Abstract

**Background:**

Child death rates are often regarded as reliable indicators for overall welfare of a country since they give insight of health accessibility and development. For planning and controlling purposes, it is important to understand which ages are at higher risks of experiencing child death as well as determinants thereof.

**Methods:**

We used the Sierra Leone DHS 2019 data which was collected using two stage sampling methods. Data collection involved interviewing women aged from 15–49 to obtain information about children they had in the past up to 2019. Age at death of child was modelled using discrete-time survival analysis with a logit link at the same time applying survey weights. The analysis also sought to estimate the determinants of child death (under-five mortality). The baseline hazard was modelled with a polynomial function.

**Results:**

Results showed that children from rural areas had significantly lower odds of dying compared with those from urban areas (odds ratio (OR) = 0.861, *p*-value = 0.0003). Children of mothers who were currently using contraceptives, and those whose mothers had been using since their last birth were at higher odds of child death compared to children whose mothers had never used contraceptives before (currently using: OR = 1.118, *p*-value =  < .0001; used since last birth: OR = 1.372, *p*-value =  < .0001). Children with no health insurance had significantly higher odds of death than those with health insurance (OR = 1.036, *p*-value =  < .0001). Children of women who were married, and of women who were formerly married were at significantly higher odds of experiencing child death than children of women who had never been in union (married: OR = 1.207, *p*-value = 0.0003; formerly married: OR = 1.308, *p*-value = 0.0009 compared to those that have never been married). Increase in the age group of mothers increases the odds of their children experiencing child death compared to mothers in their teenage years (20-29: OR = 1.943, *p*-value =  < .0001, 30-39: OR = 2.397, *p*-value =  < .0001 and >  = 40: OR = 2.895, *p*-value =  < .0001 compared to mothers in their 15-19 years).

**Conclusion:**

The study provides evidence that residing in urban areas, marital union of the mother, children having no health insurance, use of contraceptives by mother, older ages of the mother and no health insurance significantly increase the odds of child death. This points out to a possible need for improved health infrastructure to be made available to citizens in all places of delivery and more awareness on pregnancy related complications.

## Introduction

Child mortality rate (under-five mortality), as a significant national indicator of health development, has become a popular area of study with researchers aiming to establish possible measures for child mortality to be kept at an absolute minimum [1]. The United Nations Millennium Development Goal 4 (MDG4) which was targeting to reduce under-five mortality by two thirds for the period from 1990 to 2015, helped to re-focus the attention on child mortality rate [2]. By the year 2015, this MDG4 global target was still unattainable and child mortality remains acute in developing and low-middle income countries [2]. At the end of 2015, a study by The Lancet global health indicated that in low-middle income countries, 1 child in 12 dies before the age of five years, whereas in high income countries 1 child in 147 dies [3]. These figures substantiate that developing countries still require more intervention and establishments to further reduce child mortality rates. Most Sub-Saharan African countries have high child death due to low level public health [4]. In this study we will focus our analysis on the child death of Sierra Leone, a Sub-Saharan African country with one of the highest child mortality rates [4]. Sierra Leone is one of the world’s poorest countries and is experiencing numerous challenges. It has one of the highest youth unemployment rates in West Africa (of about 60%), and much of its drinking water comes from unclean sources. It is also one of Africa’s lowest literacy rates and is vulnerable to health issues such as HIV/AIDS, Malaria and Yellow fever. Approximately 81% of its population is living in poverty [5].

Even though Sierra Leone has shown improvement in reducing child mortality over the years, it is classified as one of the countries with high child and infant mortality [6, 7]. Under-five mortality rate decreased from 156 deaths per 1000 live births in the year 2013 to 122 deaths per 1000 live births in 2019. Child mortality went from 95 to 75 deaths per 1000 live births and neonatal mortality went from 39 to 31 deaths per 1000 live births [8]. These figures relatively show that the child and infant mortality rates in Sierra Leone have declined from 2013–2019, but these deaths can further be prevented provided necessary precautions and health measures are taken [6]. For that reason, it might be of importance to establish the determinants of child death. This would possibly assist in making informed decisions on which areas to improve to further lower child death.

Recent studies around the world [9–11] have determined child mortality mostly using continuous time survival models. However, in cases where data is collected in a truly discrete manner, using continuous time models gives biased estimates due to ties [12]. Methods of handling ties would then have to be adopted, [13, 14] explain extensively how ties are handled. Therefore, discrete time survival models are preferred over continuous time models because of their ability to easily allow modeling time-varying covariates and are also naturally built in a manner that addresses the issue of ties. Furthermore, the discrete time survival models do not require a hazard-related proportionality assumption that is common in continuous-time survival analysis [12]. These methods have commonly been applied to health data by [15–17] and will be considered in this study.

In addition, the standard discrete time survival model is appropriate when a simple random sample of participants was used. In an event where data analyzed was sampled using multi-stage probability sampling, using a discrete time survival model with survey weights produces more consistent estimates [18].

The objective of this study was to determine time-to-child mortality as well as factors that affect child mortality in Sierra Leone using Discrete Time Survival Analysis with survey weights.

## Statistical methods

### Discrete survival and hazard function

For a discrete-time survival analysis, the time scale is subdivided into $n$ intervals $\left\{{I}_{t}=\left[{a}_{t-1},{a}_{t}\right)\right\}$ with ${a}_{0}<{a}_{1}<...<{a}_{n}<\infty .$ Censoring and occurrence of event is assumed to happen at the end of the intervals, and the intervals do not necessarily have the same length. Let $T$ be a discrete time random variable that takes on values ${t}_{i}$ $\{i=1,..,n\}$, which denotes an occurred event in interval ${I}_{t}$ [16]. The discrete hazard function for the ${j}^{th}$ $(j=\mathrm{1,2},\dots ,M)$ individual is defined as conditional probability of failure at time interval $t$ given that the individual has survived up to time $t$ and is given by

1 $${h}_{j}\left({t}_{i}\right)=P\left(T={t}_{i}\right| T\ge {t}_{i})$$

The hazard function is always a non-decreasing function. Consequently, the survival function, which is the probability that an event of interest has not occurred by time $t$ is given by

2 $${S}_{j}\left({t}_{i}\right)=P\left(T>{t}_{i}\right)=\prod_{k=1}^{i}[1-{h}_{j}\left({t}_{k}\right)]=1-F\left({t}_{i}\right)$$

where $F\left({t}_{i}\right)$ is the cumulative probability density function, which tells us the probability of time-to-event is less than some given value ${t}_{i}$ [19, 20].

The methods of estimating the survival and hazard functions in survival analysis are broadly classified into non-parametric, semi-parametric, parametric, and discrete time methods [20]. This paper highlights one of the parametric methods which is the discrete time model as well.

To model the discrete time survival, a logistic model may be used to link the hazard function to the linear predictor, $\gamma \left(t\right)+\phi {X}_{t}$ as follows:

3 $$\mathit{logit}\{{h}_{j}\left({t}_{i}|{X}_{t}\right)\}=\mathrm{ln}\left(\frac{{h}_{j}\left({t}_{i}|{X}_{t}\right)}{1-{h}_{j}\left({t}_{i}|{X}_{t}\right)}\right)=\mathrm{ln}\left(\frac{P\left(T={t}_{i}\right| T\ge {t}_{i},{X}_{t} )}{1-P\left(T={t}_{i}\right| T\ge {t}_{i},{X}_{t} )}\right)=\gamma\left(t\right)+\phi{X}_{t}$$

So that the hazard may be obtained by solving Eq. 3 for $logit\left\{{h}_{j}\left({t}_{i}|{X}_{t}\right)\right\}$ which may be written as

4 $${h}_{j}\left({t}_{i}|{X}_{t}\right)=\frac{\mathrm{exp}(\gamma \left(t\right)+\phi {X}_{t} )}{1+\mathrm{exp}(\gamma \left(t\right)+\phi {X}_{t} )}$$

where $\gamma \left(t\right)$ is a function of time which models the baseline hazard on each time period and captures the log odds that an individual will experience the event in each time period [21] proposed different specification of the baseline hazard and $\phi$ represents the regression coefficients. Depending on the assumptions of the model, other link functions may be used such as the complementary log–log function where the underlying data was continuous but now grouped [16] or the Probit link function where normality assumptions are made [22].

### The likelihood function

To estimate the parameters $\gamma$ and $\phi$ from Eq. 4, the method of maximum likelihood can be used.

Let ${\delta }_{j}$ be an event indicator, where ${\delta }_{j}$ is 1 if an event occurs and is ${\delta }_{j}$ is 0 if an event does not occur. Then the contribution to the likelihood function by individual *j* is taken as the product of the probability of an individual to experience an event and probability of surviving beyond time ${t}_{i}$ such that,

$${L}_{j}{=h\left({t}_{i}|{X}_{t}\right)}^{{\delta }_{j }} {\left\{\prod_{i=1}^{n-1}\left[1-h\left({t}_{i}|{X}_{t}\right)\right]\right\}}^{1-{\delta }_{j}}$$

The overall likelihood for all individuals is then given by

6 $$L=\prod_{j=1}^{M}\left[{\left\{h({t}_{i}|{X}_{t}) \right\}}^{{\delta }_{j}}{\left\{\prod_{i=1}^{n-1}\left[1-h\left({t}_{i}|{X}_{t}\right)\right]\right\}}^{1-{\delta }_{j}}\right]$$

Taking the log transformation of Eq. 7 results to,

7 $$LL=\sum_{j=1}^{M}{\delta }_{J}logh({t}_{i}|{X}_{t})+\sum_{J=1}^{M}\sum_{i=1}^{n-1}\left(\left(1-{\delta }_{i}\right)\right)log\left[1-h\left({t}_{i}\right) \right]$$

Maximizing this log-likelihood and solving it with respect to the parameters will give the maximum likelihood estimates of the parameters [23, 24]. This likelihood is equivalent to that of a binary response model as used in Generalized Linear Models. This is easily implemented using readily available standard software which are designed for binary regression models [25, 12].

### Survey discrete-time survival model

Apart from the assumption of having a binary response variable, discrete-time logistic regression further makes assumptions that the explanatory variables should be independent and have equal weights [26]. In other words, it assumes that a simple random sample of the sampling units was done. This, however, was not always the case in the DHS data at hand. The ordinary discrete-time logistic model does not consider the complex nature of survey design, which may lead to incorrect statistical inference [27]. The use of discrete-time complex survey logistic model mitigates these demerits of the ordinary logistic discrete-time survival model.

In complex survey sample designs, the design includes stratification, clustering, and unequal sampling weights. A function that approximates the likelihood function in the finite sampled population and known sampling weights is constructed [28, 29]. Let assume that the finite population is partitioned into $h=\left\{\mathrm{1,2},\dots ,H\right\}$ strata, and each stratum is further divided into $l=\left\{\mathrm{1,2},\dots ,{n}_{h}\right\}$ primary sampling units (PSUs), each which is made up of $j=\left\{\mathrm{1,2},\dots ,{n}_{hl}\right\}$ secondary sampling units (SSUs), which consist of ${n}_{hlj}$ elements. Each sampling unit is associated with sampling weights which is given as the inverse of the probability of being included in the sample, denoted as

8 $${w}_{hlj}=\frac{1}{{\uppi }_{hlj}},$$

where ${\uppi }_{hlj}$ is the probability for a sampling unit to be included in the sample.

Under complex sampling design, the parameter of the regression coefficients $\phi$, from Eq. 3, are estimated by maximum pseudo-likelihood method that incorporates the sampling design and different sampling weights [28].

To model age at child death, discrete-time survival model was used with survey weights applied to account for the complex nature of the data.

### The baseline hazard

In a discrete-time survival model, time (age) is the fundamental covariate of the model. The effect of time (age) is captured in the baseline hazard. The baseline hazard, $\gamma \left(t\right),$ can be specified using distinct functions such as polynomial or piecewise constant [30, 30, 21] highlights on several ways of specifying the baseline hazard in discrete time survival models. The use of general specification of baseline hazard involves treating the discretized time variable as a categorical variable $\left[\gamma \left(t\right)={\gamma }_{1}\left(t\right)+{\gamma }_{2}\left(t\right)+{\gamma }_{3}\left(t\right)\dots \right]$ depending on the length of time interval. However, using the general constant specification does require the interval to be short. Another method that can be used when the baseline interval is large is non-parametric smoothing techniques of modelling the baseline hazard. These methods were used by [30, 25] extensively discussed how non-parametric of baseline hazard works. The effect of time is captured in the baseline hazard and omitting the time effect from the model implies that the hazard is constant over time. Using the Akaike Information Criterion (AIC) for the model selection, a baseline hazard with the smallest AIC statistic was preferred. Table 1 shows how the baseline hazards can be specified.

**Table 1:** Baseline Hazards specification

Order of Polynomial	Behavior of Logit Hazard	Number of parameters	Hazard Specification
0	Constant	1	$\gamma (t)$
1	Linear	2	$\gamma \left(t\right)+{\gamma }_{1}t$
2	Quadratic	3	$\gamma \left(t\right)+{\gamma }_{1}t+{\gamma }_{2}{t}^{2}$
3	Cubic	4	$\gamma \left(t\right)+{\gamma }_{1}t+{\gamma }_{2}{t}^{2}+{\gamma }_{3}{t}^{3}$
4	Quartic	5	$\gamma \left(t\right)+{\gamma }_{1}t+{\gamma }_{2}{t}^{2}+{\gamma }_{3}{t}^{3}+{\gamma }_{4}{t}^{4}$

### The data

This study uses data obtained from Demographic Health Surveys, which consist of data collected using different questionnaires. The data files are stored according to the nature of analysis that one intends to perform which are called recode files (i.e., Household recode, persons’ recode, women’s recode, children’s recode, etc.). Since the main objective in this study was to discover variables that are affecting child mortality rates in Sierra Leone, we used the children (kids) recode file (KR) [8].

The cross-sectional data set used in this study was obtained from the Sierra Leone Demographic Health Survey (SLDHS) 2019. The dataset is publicly available in the DHS website. Sierra Leone has five provinces, and each province is subdivided into districts. The sample for the 2019 SLDHS is a stratified sample selected using a two-stage sampling. Stratification was achieved by separating each district into urban and rural areas, which formed the enumeration areas (EA) or clusters in the first stage of stratification, 578 enumeration areas were selected according to probability proportional to the population size. From the 578 EAs, a list of households in each EA was compiled which formed the sampling frame for selection of household in second stage. A fixed number of twenty-four households were selected in second stage through equal probability systematic sampling method, which resulted to approximately 13,872 selected households [8].

The overall response rate for the 2019 SLDHS data collection was 99%, and 97% response from women aged 15 – 49 who were eligible for individual interviews. The interview included maternal history questions that helped to obtain information regarding birth dates, survival status, and age at death for children that respondents (women) gave birth to [8].

To select variables for this study, the analytical framework for the study determinants in developing countries by [31] was used. [31] proposed the incorporation of social and biological variables to study the impact that these factors have on mortality rates. As part of conceptual framework, studies by [32, 10, 11] indicated current place of residence, number of children never born, duration of breastfeeding, maternal and paternal education, household income, mother’s anemia condition, birth spacing between children and marital status to be associated with child mortality. Table 2 shows the distribution of variables used in this study, which includes some of the variables that were significant in previous studies as well as other variables selected using theoretical framework.

**Table 2:** Descriptive statistics

Covariates	Covariates Levels	n-DEAD (%)	n-ALIVE (%)	N-Total (%)
Educational Level	No education	489(4.9)	5152(52)	5641 (56.9)
	Incomplete primary education	96 (1.0)	932 (9.4)	1028 (10.4)
	Complete primary education	33 (0.3)	3.5 (344)	3.8 (377)
	Incomplete secondary education	1.8 (174)	2065 (20.9)	2239 (22.7)
	Complete secondary education	32 (0.3)	330 (3.3)	(362 (3.6)
	Higher/Tertiary education	12 (0.1)	(240) 2.4	252 (2.5)
Wealth Index	Poorest	235 (2,4)	2302 (23.3)	2537 (25.7)
	Poorer	193 (1.9)	2129 (21.5)	2322 (23.4)
	Average/Middle	186 (1.9)	1923 (19.4)	2109 (21.3)
	Richer	141 (1.4)	1599 (16.2)	1740 (17.6)
	Richest	81 (0.8)	1110 (11.2)	1191 (12)
Mother Is Currently Working	No	162 (1.6)	1960 (19.8)	2122 (21.4)
	Yes	674 (6.8)	7103 (71.8)	7777 (78.6)
Planned Pregnancy	Yes, the pregnancy was planned	818 (8.3)	8765 (88.5)	9583 (96.8)
	No, the pregnancy was not planned	18 (0.2)	298 (3.0)	316 (3.2)
Ethnicity	Creole	5 (0.1)	40 (0.4)	45 (0.5)
	Fullah	22 (0.2)	296 (3.0)	318 (3.2)
	Kono	31 (0.3)	356 (3.6)	387 (3.9)
	Limba	63 (0.6)	718 (7.3)	781 (7.9)
	Loko	12 (0.1)	172 (1.7)	184 (1.8)
	Mandingo	17 (0.2)	213 (2.2)	230 (2.4)
	Mende	295 (3.0)	3130 (31.6)	3425 (34.6)
	Sherbo	14 (0.1)	226 (2.3)	240 (2.4)
	Temme	277 (2.8)	2850 (28.8)	3127 (31.6)
	Korankoh	40 (0.4)	555 (5.6)	595 (6)
	Other	60 (0.6)	507 (5.1)	567 (5.7)
Religion	Christian	157 (1.6)	1770 (17.9)	1927 (19.5)
	Islam	677 (6.8)	7290 (73.6)	7967 (80.4)
	None/ No Religion	2 (0)	3 (0)	5 (0.05)
Marital Status	Never In Union	89 (0.9)	996 (10.1)	1085 (11)
	Married	660 (6.7)	7342 (74.2)	8002 (80.9)
	Living with Partner	40 (0.4)	384 (3.9)	424 (4.3)
	Formerly Married	47 (0. 5)	341 (3.4)	388 (3.9)
Multiple Birth	Single Birth	743 (7.5)	8729 (88.2)	9472 (95.7)
	Multiple Birth	93 (0.9)	334 (3.4)	427 (4.3)
Sex of Child	Male	470 (4.7)	4577 (46.2)	5047 (50.9)
	Female	366 (3.7)	4486 (45.3)	4852 (49)
Age of Mother	15—19 Years	61 (0.6)	605 (6.1)	666 (6.7)
	20–29 Years	380 (3.8)	4404 (44.5)	4784 (48.3)
	30–39 Years	292 (2.9)	3285 (33.2)	3577 (36.1)
	> = 40 Years	103 (1.0)	769 (7.8)	872 (19.9)
Region	Eastern	172 (1.7)	1800 (18.2)	1972 (19.9)
	Northern	171 (1.7)	2123 (21.4)	2294 (23.1)
	North Western	194 (2.0)	1688 (17.1)	1882 (19.1)
	Southern	204 (2.1)	2317 (23.4)	2521 (25.5)
	Western	95 (1.0)	1135 (11.5)	1230 (12.5)
Residential Settlement	Urban	229 (2.3)	607 (28.3)	836 (30.6)
	Rural	2797 (6.1)	6266 (63.3)	8993 (69.4)
Water Source	Piped Water	113 (1.2)	1306 (13.3)	1419 (14.5)
	Water Well	390 (4.0)	4240 (43.2)	4630 (47.2)
	Surface Water	294 (3.0)	3057 (31.1)	3351 (34.1)
	Rain Water	12 (0.1)	169 (1.7)	181 (1.8)
	Water Tankers	1 (0.0)	14 (0.1)	15 (0.15)
	Bottled Water	17 (0.2)	209 (2.1)	226 (2.3)
Place of Delivery	Other	1 (0.0)	14 (0.1)	15 (0.15)
	Home	174 (1.8)	1509 (15.2)	1683 (17)
	Government/ Public Health Sector	651 (6.6)	7346 (74.2)	7997 (80.8)
	Private Health Sector	10 (0.1)	194 (2.0)	204 (2.1)
Pregnancy Termination	No	760 (7.7)	8452 (85.4)	9212 (93.1)
	Yes	76 (0.8)	611 (6.2)	687 (7)
Contraceptive Use	Currently Using	191 (1.9)	2030 (20.5)	2221 (22.4)
	Used since last birth	50 (0.5)	353 (3.6)	403 (4.1)
	Used before last birth	120 (1.2)	1499 (15.1)	1619 (16.3)
	Never used	475 (4.8)	5181 (52.3)	5656 (57.1)
Smoking History	Has smoking history	48 (0.5)	388 (3.4)	436 (3.9)
	Doesn’t smoke	788 (8.0)	8725 (88.1)	9513 (96.1)
Health Insurance	Has health insurance	36 (0.4)	800 (8.1)	836 (8.5)
	no health insurance	289 (2.9)	8774 (88.6)	9063 (91.5)
Pregnancy Duration	< 9 Months	6 (0.1)	18 (0.2)	24 (0.3)
	9 Months	820 (8.3)	8908 (90.0)	9728 (98.3)
	10 Months	10 (0.1)	137 (1.4)	147 (1.5)
Prenatal Healthcare	No	21 (0.3)	130 (1.8)	151 (2.1)
	Yes	454 (6.2)	6772 (91.8)	7226 (98)
	Missing			2522 (25.5)
Prenatal Traditional care/birth	No	431 (5.8)	6352 (86.1)	9783 (91.9)
	Yes	44 (0.6)	550 (7.5)	594 (8.1)
	Missing			2522 (25.5)
Birth Weight	< 2 kg	12 (0.1)	117 (1.2)	129 (1.3)
	2 kg—3 kg	135 (1.4)	2838 (28.7)	2973 (30.1)
	3 kg—4 kg	95 (1.0)	2956 (29.9)	3051 (30.9)
	> 4 kg	12 (0.1)	278 (2.8)	290 (2.9)
	Not weighed at birth	582 (5.9)	2874 (29.0)	3456 (34.9)
Child Postnatal Checks	No	303 (4.1)	3620 (49.2)	53.3 (3923)
	Yes	170 (2.3)	3265 (44.4)	3435 (46.7)
	Missing			2541 (25.7)
Preceding Birth Interval	1–12 Months	26 (0.3)	94 (0.9)	120 (1.2)
	13–24 Months	180 (1.8)	974 (9.8)	1154 (11.6)
	25–48 Months	305 (3.1)	3415 (34.5)	3720 (37.6)
	49–60 Months	45 (0.5)	899 (9.1)	944 (9.6)
	> 60 Months	82 (0.8)	1562 (15.8)	1644 (16.6)
	No Previous Birth	198 (2.0)	2119 (21.4)	2317 (23.4)

**Fig. 1:**
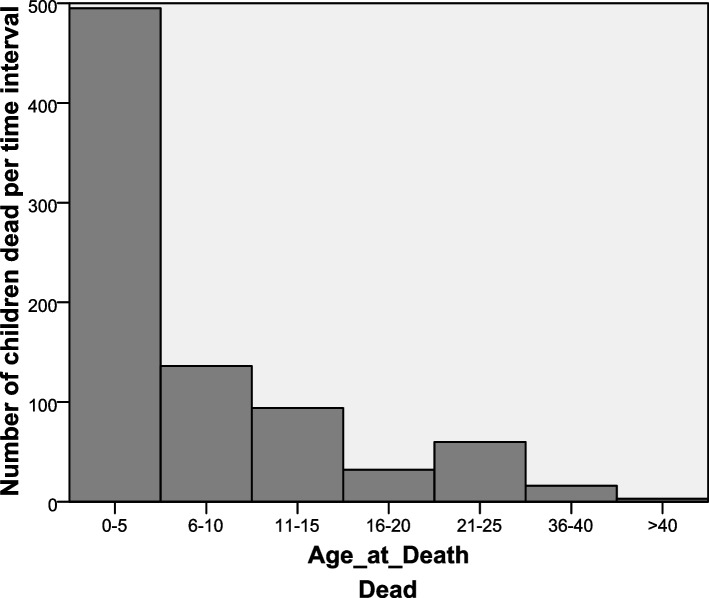
Distribution of death by age

Figure 1 shows that the distribution of death by age. It is evident that majority of child death occur in between month 0 and month 5. the distribution of number of deaths by time is positively skewed.

The proportion of children who died before the age of 60 months in Sierra Leone was 8.4% and 91.6% was reported to be alive. Descriptive statistics results from Table 2 indicate that there are 6.1% of the children residing in rural areas in Sierra Leone who died before turning 60 months while 2.3% of the children in urban areas died before the age of 60 months. Majority of children who died had mothers with no education 4.9% and 0.1% of children who died had mothers with higher education. For children with no health insurance, 8.1% of them died while 0.4% of children with health insurance died. There was 5.8% and 4.1% of children who died and did not receive prenatal and postnatal care, respectively.

### Statistical software

The statistical analysis in this paper was done using the statistical software SAS Enterprise Guide 7.1 and SAS On Demand (SAS Studio). To fit the discrete-time model, we use the *PROC LOGISTIC* procedure in SAS. However, this fits the ordinary discrete-time logistic model and computes statistics with assumption that the sample is chosen using simple random sampling methods [33]. Since the SLDHS is a survey data with survey weights, using a *PROC SURVEYLOGISTIC* model is more appropriate as it allows for specification of survey weights, stratification and clusters that are included in the sample design [33].

## Results and discussion

**Fig. 2:**
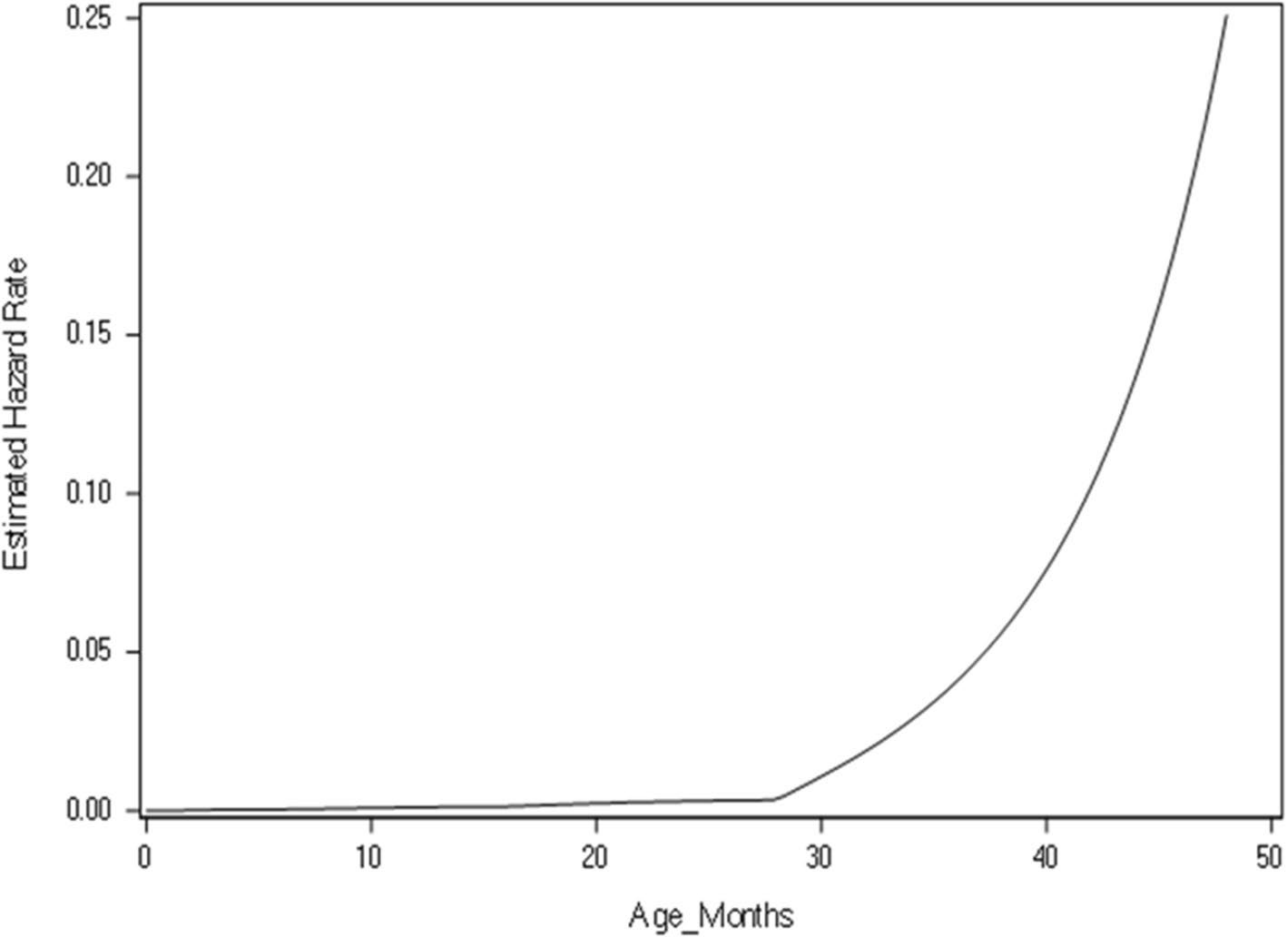
Baseline Hazard Plot

Since the age interval in the study has sixty intervals, the parametric method of fitting the baseline hazard in a discrete time survival model involved fitting polynomial functions of the intercept $\gamma \left(t\right)$ from the model. In this study we fitted four distinct functions of the baseline hazard and according to the results in Table 3. Based on the model with smallest AIC it can be concluded that modelling the baseline hazard with cubic polynomial was best fit. Figure 2 shows the baseline hazard, the hazard of child death was steadily constant at about zero from month 0 to month 28 and began to exponentially rise after month 28.

**Table 3:** Baseline Hazard AIC Results

Baseline Hazard	AIC
Constant	62,270.190
linear	56,377.274
Quadratic	56,273.655
Cubic	53,556.935
Logarithmic	56,471.587

### Model results

Table 4 displays estimates results from two models, namely, basic discrete-time model, and the survey discrete-time model.

**Table 4:** Results from the two Fitted Discrete time Models

	**BASIC DISCRETE MODEL**		**SURVEY DISCRETE MODEL**	
**VARIABLE**	**ODDS RATIO**	*P*-VALUE	**ODDS RATIO**	***P*** **-VALUE**
**PREGNANCY DURATION (REF = 9 Months)**				
< 9 Months	0.989	0.9704	0.593	0.1418
10 Months	1.002	0.9870	0.952	0.7129
**REGION (REF = Western)**				
Eastern	1.039	0.5509	1.108	0.1271
Northern	1.151	0.0151*	1.216	0.0016*
North-Western	1.016	0.7915	1.056	0.3945
Southern	1.112	0.0981	1.193	0.0104*
**RESIDENTIAL SETTLEMENT (REF = Urban)**				
Rural	0.880	0.0062*	0,861	0.0003*
**RELIGION (REF = No religion)**				
Christian	0.535	0.4000	0.476	0.0917
Islam	0.545	0.4146	0.480	0.0952
**ETHNICITY (Ref = Other)**				
Creole	1.103	0.6183	1.012	0.9517
Fullah	0.929	0.4139	0.962	0.6907
Kono	0.962	0.6690	0.952	0.5537
Limba	1.081	0.2798	1.084	0.2530
Loko	1.163	0.1706	1.235	0.1403
Mandingo	0.930	0.4663	0.874	0.1436
Mende	0.999	0.9938	0.973	0.6761
Sherbo	0.871	0.1853	0.905	0.3253
Temme	0.937	0.2798	0.954	0.4457
Korankah	0.903	0.1901	0.946	0.4960
**EDUCATIONAL LEVEL (REF = No education)**				
Incomplete primary	0.966	0.4342	0.966	0.4196
Complete primary	1.053	0.4493	1.051	0.4573
Incomplete secondary	0.981	0.6014	0.963	0.3463
Complete secondary	1.014	0.8554	1.083	0.3788
Higher	1.091	0.3196	1.102	0.2574
**WEALTH INDEX (REF = Poorest)**				
Poorer	1.020	0.5897	1.030	0.4289
Middle	1.028	0.4879	1.031	0.4351
Richer	0.947	0.3439	0.946	0.3185
Richest	0.994	0.9338	0.983	0.8101
**PREGNANCY TERMINATION (REF = Yes, previously)**				
No, Has Never Terminated Before	0.989	0.8222	1.004	0.9371
**CONTRACEPTIVE USE (REF = Never used)**				
Currently using	1.109	0.0011*	1.118	0.0003*
Used since last birth	1.348	< .0001*	1.372	< .0001*
Used before last birth	1.024	0.5577	1.037	0.4060
**SMOKING HISTORY (REF = Does not smoke)**				
Has smoking history	1.013	0.8392	1.036	0.5250
**HEALTH INSURANCE (REF = Has health insurance)**				
Has no health insurance	1.291	0.0005*	1.377	< .0001*
**MARITAL STATUS (REF = Never in union)**				
Married	1.199	0.0001*	1.207	0.0003*
Living with a partner	1.068	0.3814	1.088	0.3277
Formerly Married	1.236	0.0052*	1.308	0.0009*
**CURRENTLY WORKING (REF = Yes, employed)**				
No, unemployed	0.952	0.1540	0.947	0.1303
**MULTIPLE BIRTH (REF = Multiple birth)**				
Single birth	1.079	0.2819	1.031	0.7535
**SEX OF CHILD (REF = Female)**				
Male	0.989	0.6724	0.996	0.8985
**PRENATAL HEALTHCARE (REF = No, received no healthcare)**				
Yes, received healthcare	0.901	0.2515	0.896	0.2919
**PRENATAL TRADITIONAL CARE (REF = No traditional care)**				
Yes, received traditional care	0.959	0.3721	0.896	0.2919
**PLANNED PREGNANCY (REF = No)**				
Yes	1.142	0.0688	1.135	0.1080
**Birth Weight (REF = less than 2 kg)**				
2 kg—3 kg	1.309	0.0217*	1.238	0.0586
3 kg—4 kg	1.009	0.7751	1.015	0.7039
More than 4 kg	1.077	0.3334	1.000	0.9990
Not weighed at birth	0.898	< .0001*	1.379	< .0001*
**PLACE OF DELIVERY (REF = Private health sector)**				
Other	0.815	0.5158	0.786	0.4392
Home	1.307	0.0060*	1.289	0.0395*
Public sector	1.302	0.0039*	1.289	0.0267*
**CHILD POSTNATAL CHECKUPS (REF = No, check-ups were not done)**				
Yes, check-ups were done	1.011	0.6725	1.012	0.6885
**WATER SOURCE (REF = Piped water)**				
Water well	0.968	0.4180	0.952	0.2343
Surface water	0.963	0.4038	0.942	0.1767
Rainwater	0.895	0.2698	0.898	0.3853
Water tankers	0.806	0.5020	0.708	0.2174
Bottled water	0.878	0.1768	0.974	0.7946
**AGE OF MOTHER (REF = 15–19 Years)**				
20–29 Years	1.941	< .0001*	1.943	< .0001*
30–39 Years	2.357	< .0001*	2.397	< .0001*
> = 40 Years	2.891	< .0001*	2.895	< .0001*
**PRECEDING BIRTH INTERVAL (REF = > 60 Months)**				
0, No previous pregnancy	1.383	< .0001*	1.424	< .0001*
1–12 Months	1.094	0.4556	1.039	0.7433
13–24 Months	1.263	< .0001*	1.295	< .0001*
25–48 Months	1.090	0.0257*	1.125	0.0040*
49–60 Months	0.960	0.4235	0.961	0.4710

Key: *P-Value < 0.05, indicates statistically significant covariates

Table 4 illustrates how the fitted discrete-time models produce partially consistent results in terms of statistical significance of explanatory variables, the significance of the explanatory variables was tested under $$5\%$$ level of significance. Results from the survey discrete-time logistic model indicate that children whose mothers were from Northern and Southern region in Sierra Leone were at significantly higher odds of experiencing child death than those children whose mothers were from the Western region. $$(OR = 1.216, p-value=0.0016; OR = 1.193, p-value=0.0104,$$ respectively $$)$$

Output further highlights that children whose mothers were from rural areas were at significantly lower odds of child mortality compared to children whose mothers were from urban areas$$(OR = 0.861, p-value=0.0008)$$. Use of contraceptives was also a significant predictor of child death. It is evident that children whose mothers were currently using contraceptives and those whose mothers who had been using them since their previous pregnancy $$(OR=1.118, p-value=0.0003; OR=1.372, p-value=<.0001,$$ respectively $$)$$ were more likely to experience child death than those whose mothers had never used contraceptives.

Health Insurance had positive relationship with child death in Sierra Leone. Children whose mothers had no health insurance had significantly higher odds of dying $(OR=1.337; p-value=<.0001)$ than children whose mothers had health insurance. Certain levels of marital status were statistically significant predictors of child death. It is further notable how children of married, and formerly mothers were at significantly higher odds $\left(OR= 1.207, p-value=0.0003; OR=1.308, p-value=0.0009, \mathrm{respectively}\right)$ of experiencing child death than children whose mothers had never been in union.

Place of delivery variable indicates that children that were delivered at home and public health sectors were at significantly higher odds of dying$$(OR=1.298, p-value=0.0395; OR=1.262, p-value=0.040$$, respectively $$)$$ than children that were delivered at private health sectors. Increase in mothers age illustrates an increase in the odds of having children more prone to child death.$$(20-29: OR=1.943, p-value=<.0001;30-39: OR=2.397, p-value=<.0001;>40 age: OR=2.895, p-value=<.0001$$; respectively$$)$$. The preceding birth interval was also a statistically significant predictor of child death, it is notable children whose mothers have low interval birth spacing are at significantly higher odds of experiencing child death that children whose mothers have higher interval birth spacing$$(no previous birth:OR=1.424, p-value=<.0001;1-12 months:OR=1.039, p-value=0.7433;13-24 months:OR=1.295, p-value=<.0001;25-48 months:OR=1.125, p-value=0.0040;49-60 months:OR=0.961, p-value=0.4710$$).

## Discussion

The aim of this study was to model age at child death and determine factors that are associated with child death in Sierra Leone using the 2019 DHS data. This analysis was done using discrete-time survival models, and discrete-time survival with survey weights. From the fitted models, the discrete-time survival with survey weights has a slightly higher predictive power than the basic discrete-time survival model. Therefore, the analysis and interpreted results are based on this model. It was discovered that the median age of experiencing child death in at 16 months from birth and it behaved in a cubic polynomial, as indicated by the fitted baseline hazard.

Place of residence was a significant predictor of age at child death, where children residing in rural area were at lower risk of experiencing death compared to those residing in urban areas. Initiatives to improve health service and affordability, such as Free Health Care Initiative (FHCI) and Rural Health Care Initiative (RHCI), may have had a significant impact on lowering child death rates especially in rural areas. Most of the studies conducted prior to 2019, [10, 34], contradict findings from our study with regards to place of residence as they discovered that children in rural areas are more prone to experiencing child death than those in urban areas.

According to our study, the use of contraceptives was significantly associated with age at child death, for mothers who had used them historically and those that were currently using them. However, other existing literature about long term effects of contraceptives, [35–37], revealed that contraceptives do not have adverse effects on children. A study by [38], however, shows results that are consistent with what was discovered in this study that contraceptive use is associated with increased risk of child mortality.

Other significant factors which show consistent results with what was discovered by other authors include: marital status, preceding birth interval and mother’s age. The cross-sectional nature of the SLDHS makes it difficult to discover the actual causes of the child death [39]. Birth spacing has remained a significant predictor of child death over the years around this research topic. Our study showed that birth spacing of less than two years significantly increased the odds of child death. In addition, women who had never used contraceptives in Sierra Leone were married. This may result in short interval birth spacing as majority of married women may not be practicing family planning. Increase in birth spacing reduces risk of child death as the children are not compelled to stopping breastfeeding too soon, which has been said to affect development and growth of the child [40].

The study done by [10], where time to child death was modeled using continuous time models (Cox proportional hazard model), showed how postnatal visits reduces the hazard of child death, however in this study child postnatal checkups was not a significant predictor of child death. Gender, religion, and wealth were used as a covariate in this study and results show that they are not significant predictors of age to child death. These results are consistent with the findings from [10]. In [11]’s study there is evidence of marital status and educational level being statistically significant covariates of modeling child mortality. Similarly, in this study, some levels of marital status (married and formerly married) are significant predictors of child death. Many authors have produced research about child mortality in various parts of the world, however, there is very few available literature which focus on the time to child mortality. Therefore, this study will have significant contribution to literature as it stretches the study of child mortality to also studying how age is plays a role in child death.

## Conclusion

In this study we modelled age at child death in Sierra Leone using discrete time survival with survey weights. Results show that the baseline hazard of child death is behaving in a cubic polynomial shape. Furthermore, we see how having health insurance reduced the odds of experiencing child death. Mother having children at older age also increased the odds of their children to be prone to child death. Another variable that gave significant findings is the place of delivery, as it shows how those who deliver at home and public sectors had greater odds of having their children dying before the age of 5 years than those that may have delivered at private health sectors. This may be due to lack of proper infrastructure, sufficient health services or high volumes of people dependent on limited available health care.

The results obtained from this study are important and may assist the government of Sierra Leone in policy making decisions, particularly with regards to how the country can achieve Sustainable Development Goals and maintain low child mortality rates. Along with other variables that may be significant predictors of child death, the ones identified in this study can formulate guidance on areas that require stringent monitoring to ensure that Sierra Leone reaches the sustainable development goals.

## Data Availability

The datasets supporting the results and conclusion in this study are publicly available from the DHS program https://dhsprogram.com/methodology/survey/survey-display-545.cfm

## Abbreviations

AIC: Akaike Information Criterion
DHS: Demographic Health Survey
MDG4: Millennium Development Goal 4
OR: Odds Ratio
PSU: Primary Sampling Units
SLDHS: Sierra Leone Demographic Health Survey
SSU: Secondary Sampling Units
